# Growth hormone secretagogue receptor is important in the development of experimental colitis

**DOI:** 10.1186/s13578-015-0002-5

**Published:** 2015-03-21

**Authors:** Zhen-ze Liu, Wei-gang Wang, Qing Li, Miao Tang, Jun Li, Wen-ting Wu, Ying-han Wan, Zhu-gang Wang, Shi-san Bao, Jian Fei

**Affiliations:** School of Life Science and Technology, Tongji University, Shanghai, 200092 China; Shanghai Research Centre for Model Organisms, Shanghai, 201203 China; Discipline of Pathology, Bosch Institute and School of Medical Sciences, University of Sydney, Sydney, NSW 2006 Australia

**Keywords:** Growth hormone secretagogue receptor (GHSR), Ghrelin, Colitis, Macrophages, D-lys^3^-GHRP6

## Abstract

**Background:**

Growth hormone secretagogue receptor (GHSR) and its ligand, ghrelin, are important modulators in weight control and energy homeostasis. Recently, ghrelin is also involved in experimental colitis, but the role of GHSR in the development of colitis is unclear. The aim was to examine the underlying mechanism of GHSR in IBD development.

**Methods:**

The temporal expression of GHSR/ghrelin was determined in dextran sulphate sodium (DSS) induced colitis in Wt mice. The severity of DSS induced colitis from GHSR^−/−^ and WT mice was compared at clinical/pathological levels. Furthermore, the function of macrophages was evaluated *in vivo* and *in vitro*.

**Results:**

Lack of GHSR attenuated colitis significantly at the clinical and pathological levels with reduced colonic pro-inflammatory cytokines (P < 0.05). This is consistent with the observation of less colonic macrophage infiltration and TLRs expression from DSS-treated GHSR^−/−^ mice compared to WT mice (P < 0.05). Furthermore, there was significantly reduced pro-inflammatory cytokines in LPS-stimulated macrophages *in vitro* from GHSR^−/−^ mice than WT mice (P < 0.05). Moreover, D-lys^3^-GHRP6 (a GHSR antagonist) reduced LPS-induced macrophage pro-inflammatory cytokines from WT mice *in vitro*.

**Conclusions:**

GHSR contributes to development of acute DSS-induced colitis, likely *via* elevated pro-inflammatory cytokines and activation of macrophages. These data suggest GHSR as a potential therapeutic target for IBD.

**Electronic supplementary material:**

The online version of this article (doi:10.1186/s13578-015-0002-5) contains supplementary material, which is available to authorized users.

## Background

Growth hormone secretagogue receptor (GHSR) is a G protein-coupled receptor with broad distributions in diverse tissues. Ghrelin, the ligand of GHSR, is predominately secreted by the gastrointestinal system. Ghrelin stimulates the pituitary gland to release growth hormone (GH) [1] and plays a critical role in maintaining energy homeostasis and body weight *via* regulating food intake [2]. Recent evidence suggests that ghrelin also regulates immune responses [3,4], with anti-inflammatory properties on human monocytes and T cells *in vitro* [5] and therapeutic functions in a number of animal models, such as endotoxic shock and gastrointestinal disease [6].

The role of ghrelin/GHSR signaling in the gastrointestinal immunity is controversial, although it has been demonstrated that ghrelin is anti-inflammatory [3]. Ghrelin increases IL-8 (neutrophil chemotactic factor) production *via* activating NF-κB pathway in human GHSR transgenic colonic epithelial cells [7].

Inflammatory bowel disease (IBD) is a chronic uncontrolled inflammation of the intestinal mucosa, which can be classified into Crohn’s disease (CD) and ulcerative colitis (UC) [8]. Despite extensive research over decades, the precise underlying mechanism remains to be fully understood. It is believed that the pathogenesis of IBD is associated with the dysregulated immune response to intestinal bacterial flora, with contributions from both genetic predisposition and environmental factors [9]. A recent study reported that the severity of DSS-induced colitis is attenuated in ghrelin^−/−^ mice, but such attenuation is reversed by exogenous ghrelin [10], which suggests ghrelin is involved in the regulation of IBD. However the possible role of GHSR in development of IBD is still not fully understood. Considering the close biological linkage between ghrelin and GHSR, it is reasonable to hypothesise that GHSR contributes to the pathogenesis of colitis.

DSS-induced colitis was used to explore the pathogenesis of GHSR in colitis, the severity of disease was determined at both clinical and pathological levels from GHSR knockout (GHSR^−/−^) mice and their WT littermates. Furthermore, macrophages are important in acute DSS-induced colitis [11], and ghrelin is also considered to regulate the activity of macrophages [12]. Therefore, the production of pro-inflammatory mediators by GHSR^−/−^ macrophages following LPS stimulation was examined in vitro and compared to macrophages from Wt mice. Finally, the GHSR pathway was validated by receptor blockade in LPS-stimulated macrophages, using an antagonist of GHSR, D-lys3-GHRP6 (DLS). Our study may provide useful data to support the development of new therapeutic options for inflammatory bowel disease.

## Results

### Temporal expression pattern of GHSR/ghrelin

**Figure 1:**
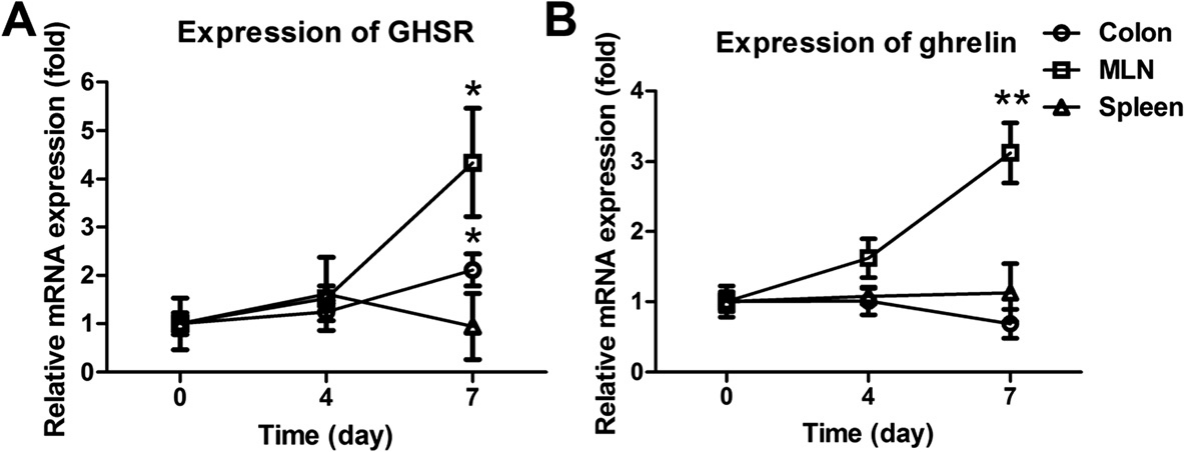
**Temporal expression patterns of GHSR/ghrelin during acute colitis induced by DSS.** C57/BL6 mice were given 2% DSS aqueous solution instead of normal drinking water for 7 days. Mice were sacrificed at day 0, 3, 7 and 11; colons, MLNs and spleens were harvested for gene expression analysis. The RNA levels of GHSR **(A)** and ghrelin **(B)** were quantified by rRT-PCR. Each point represents the mean ± SEM; *p < 0.05 and **p < 0.01.

The temporal expression patterns of GHSR and ghrelin during DSS challenge were examined in colon, mesenteric lymph nodes (MLN) and spleen at 3 time points (day 0, 4 and 7). Our results demonstrated that GSHR mRNA expression in MLN or colon, but not spleen, were increased by ~3 (*p* < 0.05) or ~1 fold (*p* < 0.05) at day 7 DSS challenge, respectively (Figure 1A).

Moreover, there was constitutive level of ghrelin mRNA in colon, MLN and spleen. Splenic ghrelin mRNA was induced by ~2 fold (*p* < 0.01) in MLN at day 7 from DSS-challenged WT mice. However, no obvious change of ghrelin expression was observed in colon or spleen following 7 days DSS treatment (Figure 1B).

### Lack of GHSR attenuated DSS-induced acute colitis

**Figure 2:**
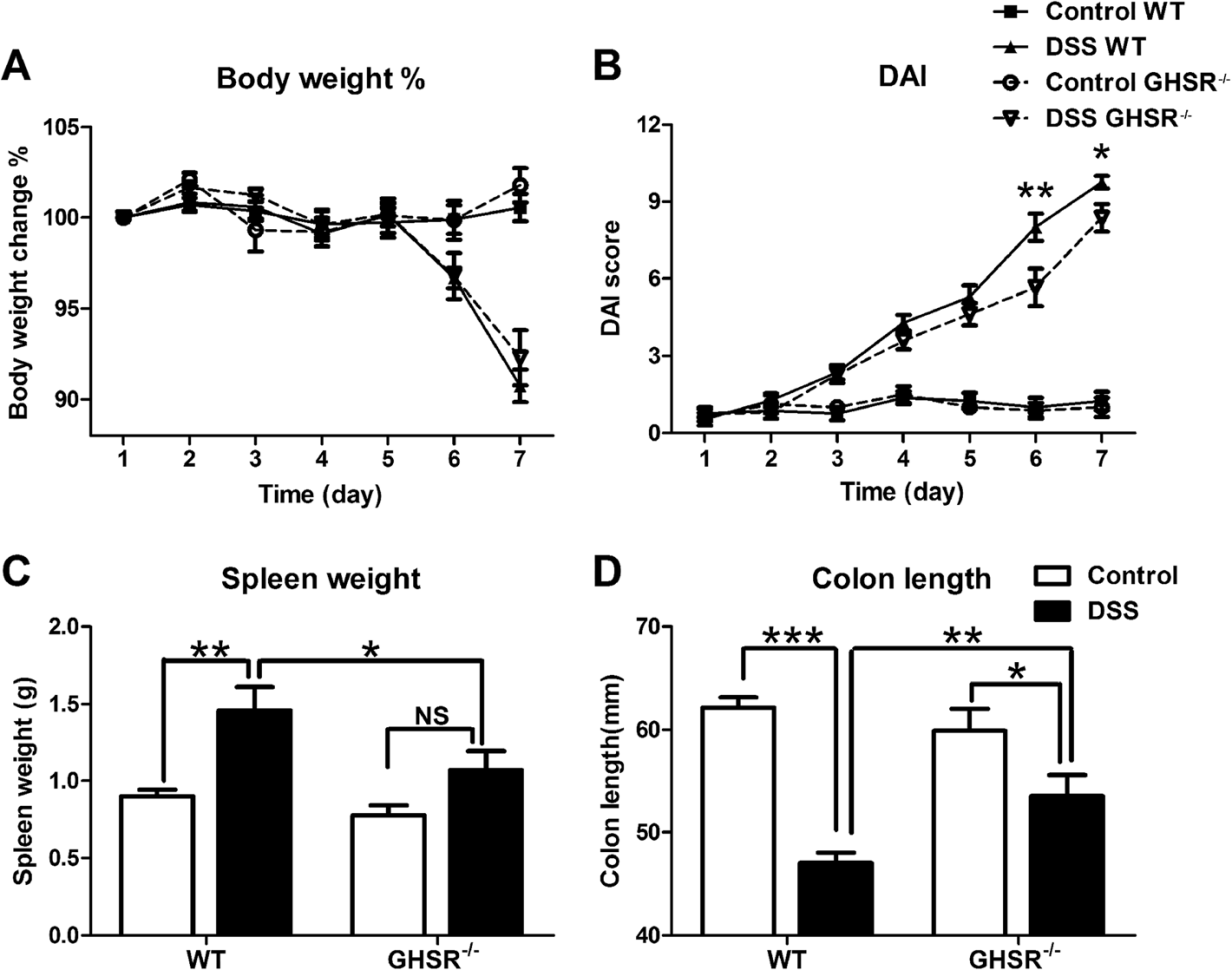
**Symptoms of acute DSS-induced colitis in WT and GHSR** ^**−/−**^ **mice.** Acute colitis was induced in WT and GHSR^−/−^ mice by drinking 2% DSS for 7 days. Body weight change **(A)** and DAI score **(B)** which consist of weight loss, diarrhoea and faecal blood was given to each group daily to monitor the disease development. On day 7, mice were sacrificed, and disease evaluations were given at macroscopic level, including spleen weights **(C)** and colon shortening **(D)**. Each point or bar represents the mean ± SEM; NS: no significance, *p < 0.05, **p < 0.01, and ***p < 0.005.

There was no obvious body weight change or clinical as well as histopathological abnormalities in mock challenged GHSR^−/−^ and WT mice, suggesting that deficiency of GHSR does not cause spontaneous inflammation in the gastrointestinal system. Significant body weight loss was observed on days 6 and 7 in DSS challenged GHSR^−/−^ and WT mice, compared to that of mock challenge (*p* < 0.05) (Figure 2A). However there was no significant difference between WT and GHSR^−/−^ mice with DSS challenge at any time point. Disease activity index (DAI) score was used to evaluate the clinical severity of colitis, referring to three parameters, i.e. body weight loss, diarrhoea and faecal blood as descried previously [13]. The body weight change was monitored twice day, each group there were 6 mice. Thus the mean and SD of the bodyweight change were calculated. The DAI score was gradually increased from day 1 to day 7 in both DSS-treated GHSR^−/−^ and WT mice. There was significantly higher DAI in WT with DSS challenge at days 6 (*p* < 0.01) or 7 (*p* < 0.05), compared to GHSR^−/−^ mice (Figure 2B), suggesting attenuation of clinical signs in DSS-treated GHSR^−/−^ mice.

Splenic weight at day 7 was measured as it reflects systemic immunity. There was no difference in splenic weight between WT and GHSR^−/−^ mice without challenge. A ~2-fold increase of splenic weight was observed in WT mice with DSS challenge, compared to mock challenged (*p* < 0.01), but no significant different splenic weight was detected in GHSR^−/−^ mice between DSS and mock treatment. Moreover, splenic weight from DSS-challenged WT was 40% higher than that of GHSR^−/−^ mice (*p* < 0.05) (Figure 2C).

Colon length is an objective macroscopic criteria in determining severity of colitis [13,14]. No significant difference in colon length was observed between GHSR^−/−^ and WT mice without challenge. However, there was ~25% (*p* < 0.001) or ~10% (*p* < 0.05) reduction for the WT or GHSR^−/−^ mice, respectively, with DSS 7 day treatment, compared to unchallenged counterparts. In addition, colon length was significantly shortened in WT compared to that of GHSR^−/−^ mice following 7 day DSS challenge (*p* < 0.05) (Figure 2D).

**Figure 3:**
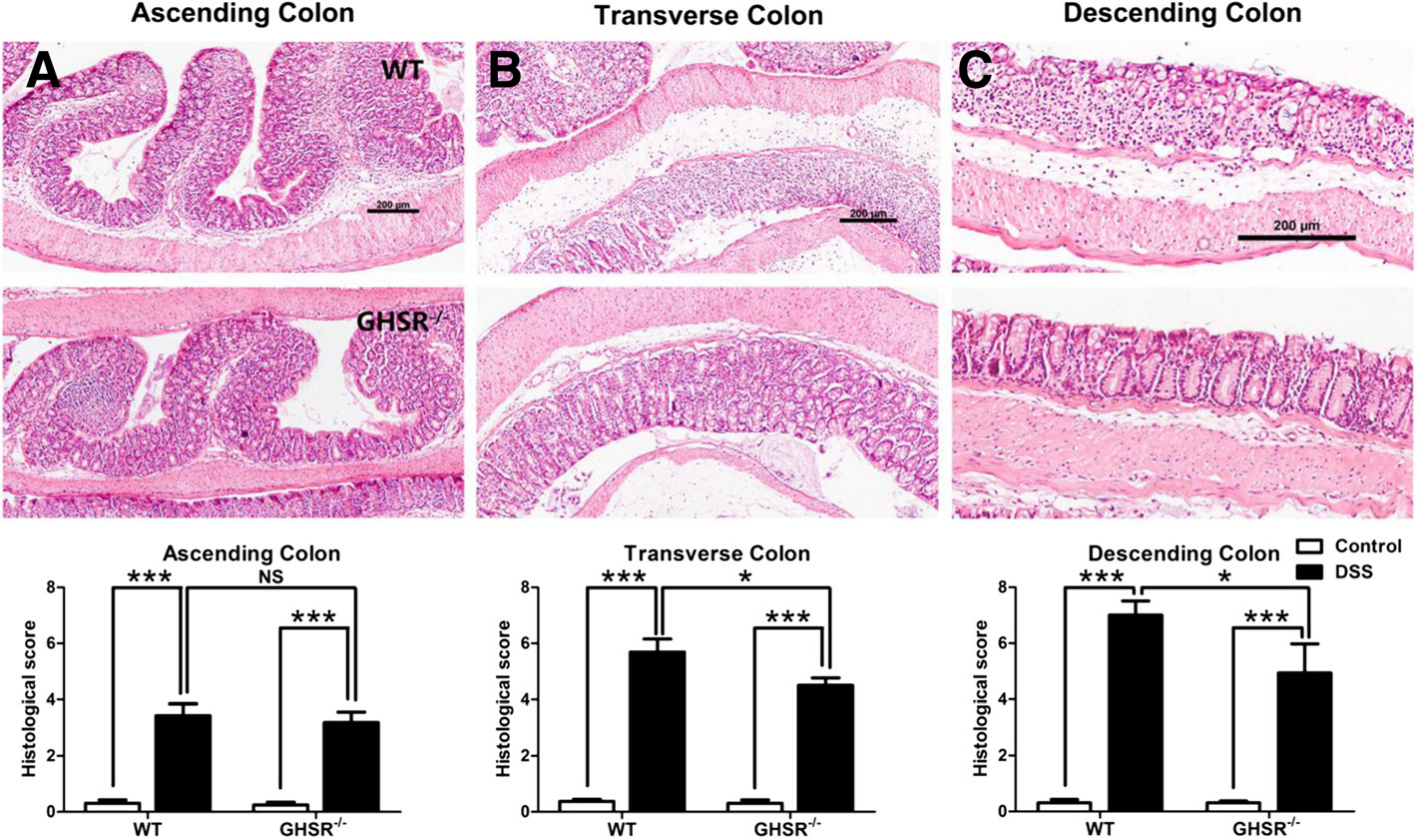
**Histopathological analysis for the acute DSS-induced colitis in WT and GHSR** ^**−/−**^ **mice.** Colons from DSS-treated WT and GHSR^−/−^ mice were collected and prepared for H&E staining. The mucosal damage/inflammation in ascending **(A)**, transverse **(B)** and descending portions **(C)** were estimated using a histological scoring system. Each bar represents the mean ± SEM; NS: no significance, * *p* < 0.05 and *** *p* < 0.005.

Histopathology score, including intestinal inflammation and crypt damage, was applied to determine the severity of colitis, as described [13]. There was no obvious colonic damage from both GHSR^−/−^ and WT mice without challenge. Severe inflammation was observed in the colons from both GHSR^−/−^ and WT mice following 7 days DSS treatment, showing epithelial ulceration, crypt damage, goblet cell loss and infiltration of inflammatory cells. Additionally, in agreement with previous studies [13], the damage/inflammation in transverse/descending colon (Figure 3A, B) was much more severe than had occurred in the ascending colon (Figure 3C).

Significantly higher histopathological score was observed in transverse (WT: 5.7 ± 0.5, GHSR^−/−^: 4.5 ± 0.5, *p* < 0.05) and descending colons (WT: 7.0 ± 0.5, GHSR^−/−^: 4.9 ± 1.0, *p* < 0.05) from DSS-treated WT, compared to GHSR^−/−^ mice. However, there was no significant difference in histopathological score in ascending colon between DSS-treated WT and GHSR^−/−^ mice (Figure 3A, B, C).

### Lack of GHSR inhibits the production of cytokines

**Figure 4:**
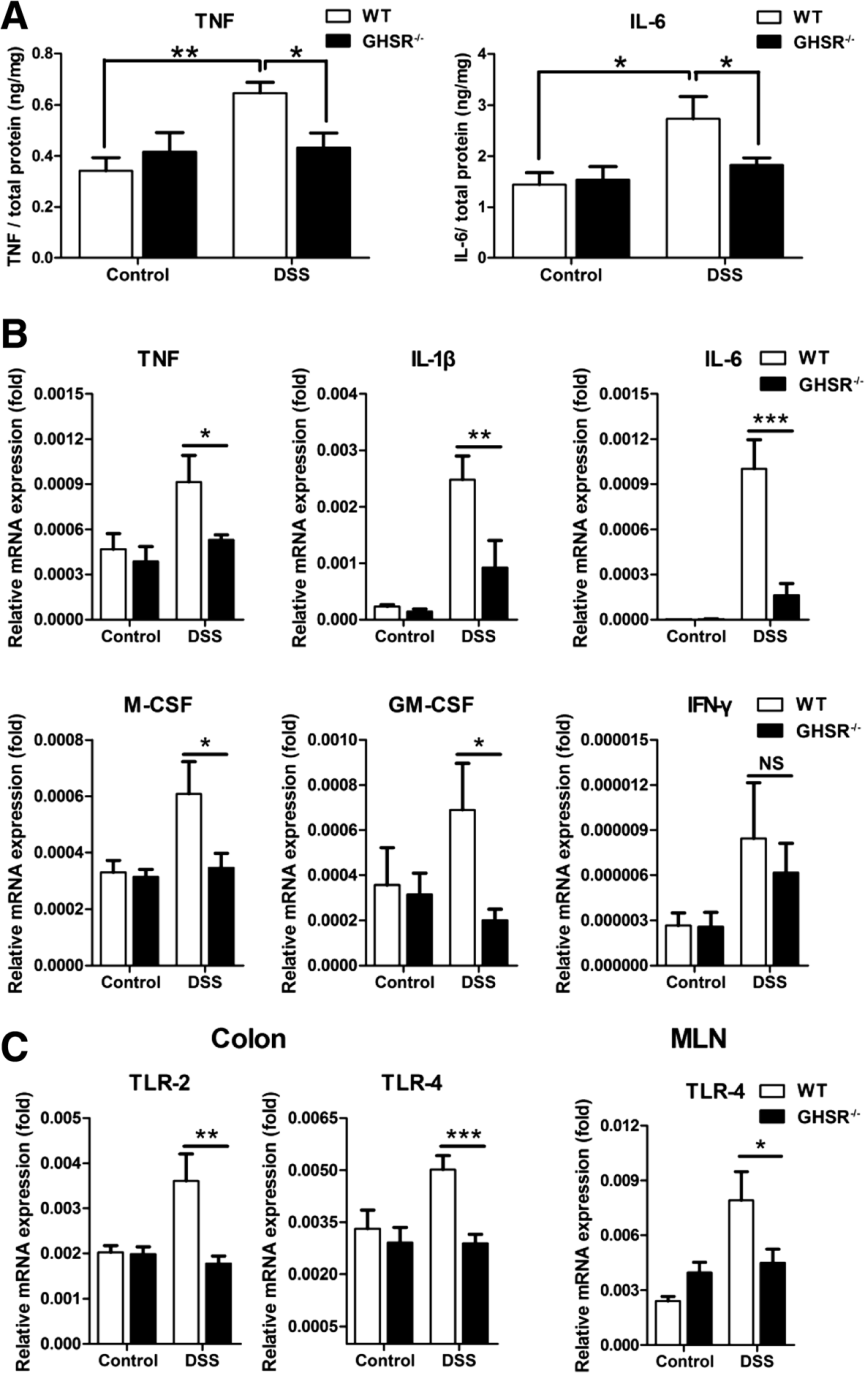
**Productions of pro-inflammatory cytokines and expressions of TLRs.** Colons from mock or DSS-treated WT and GHSR^−/−^ mice were collected, and the production of pro-inflammatory cytokines TNF and IL-6 were detected by ELISA. The final results were expressed as the radio of cytokine concentration to total protein content **(A)**. Moreover, the RNA levels of TNF, IL-1β, IL-6, M-CSF, GM-CSF and IFN-γ were also examined in colons **(B)**. Finally, the innate immune receptors of TLR-2 and TLR-4 were quantified in colon and MLN as well respectively **(C)**. Each bar represents the mean ± SEM; *p < 0.05, **p < 0.01, and ***p < 0.005.

There was constitutive level of colonic TNF from both GHSR^−/−^ and WT mice without significant difference, as measured by tissue ELISA. Colonic TNF was significantly upregulated in DSS-challenged WT mice (~2 fold, *p* < 0.01), but not GHSR^−/−^ mice, compared to mock treated mice. Furthermore, colonic TNF was significantly higher in DSS-challenged WT compared to that of GHSR^−/−^ mice (*p* < 0.05) (Figure 4A). A similar pattern was observed in colonic IL-6 production, which was markedly increased in DSS-challenged WT mice compared to mock treated WT mice (~2 fold, *p* < 0.05) or DSS-treated GHSR^−/−^ mice (~2 fold, *p* < 0.05) (Figure 4A).

Colonic mRNA levels of TNF, IL-1β, IL-6, IFN-γ, M-CSF and GM-CSF were quantified, using rRT-PCR. There were constitutive levels of colonic TNF, IL-1β, IL-6, IFN-γ, M-CSF and GM-CSF without significant difference between GHSR^−/−^ and WT mice. The expressions of TNF, IL-1β, IL-6, M-CSF and GM-CSF were up-regulated by 11 fold, (p < 0.01), 348 fold (p < 0.05), 2 fold (p < 0.05), 2 fold (p < 0.05), 2 fold (p < 0.05), respectively in WT mice following DSS challenge, excepted for and IFN-γ (no significance). These cytokines were also up-regulated significantly in GHSR^−/−^ mice following DSS challenge, but TNF, IL-1β, IL-6, M-CSF or GM-CSF for GHSR^−/−^ mice were 40%, (p < 0.05), 60%, (p < 0.01), 80%, (p < 0.05), 40%, (p < 0.05), 70%, (p < 0.05) lower compared to WT mice following DSS challenge (Figure 4B).

### Lack of GHSR decreases TLRs expression

TLR-2 and TLR −4 are two key molecules involved in triggering innate immunity, which plays an important role in DSS-induced acute colitis [9,11]. There was a constitutive level of colonic TLR-2 or TLR-4 expression for both GHSR^−/−^ and WT mice without significant difference. The expression of colonic TLR-2/4 was 1.8 (*p* < 0.05), or 1.5 (*p* < 0.01) fold increased, respectively, following 7 days DSS challenge in WT mice. However, there was no obvious change of colonic TLR-2 or TLR-4 for DSS-challenged GHSR^−/−^ mice; which was significantly lower than WT expression of TLR-2 (*p* < 0.01) or −4 (*p* < 0.005). Moreover, a similar pattern was observed in MLN expression of TLR-4, but not TLR-2. GHSR^−/−^ showed a ~40% lower expression of TLR-4 than WT (*p* < 0.05) following 7 days DSS challenge (Figure 4C).

### Lack of GHSR reduces macrophage infiltration

**Figure 5:**
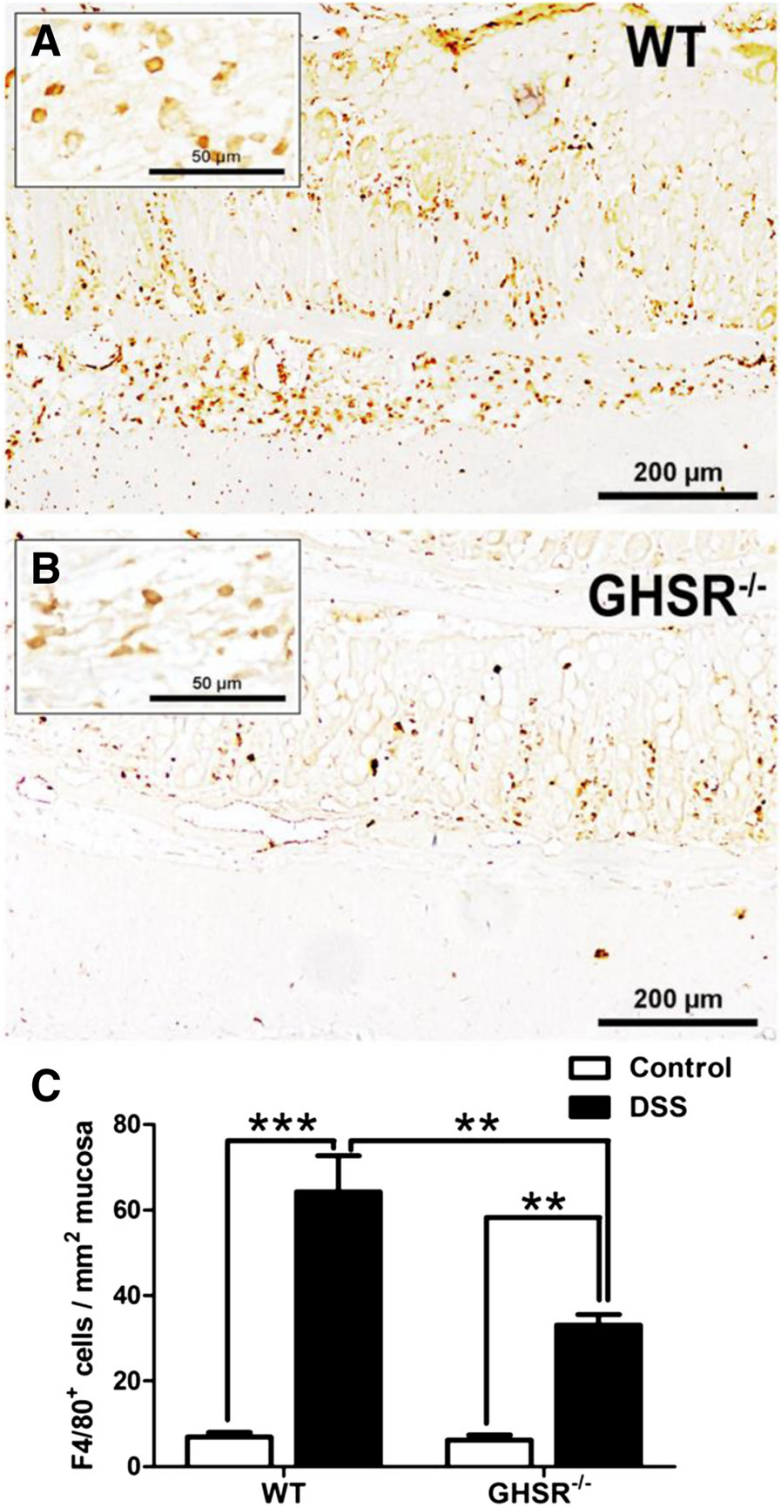
**Infiltration of macrophages in DSS-treated WT and GHSR** ^**−/−**^ **colons.** Colonic infiltrating macrophages were examined by immunohistochemical staining of F4/80. More F4/80^+^ cells were observed in the submucosal region of inflamed WT colon **(A)** than GHSR^−/−^ **(B)**. In addition, the numbers F4/80^+^ cells in the mucosal and submucosal layer were counted, and the final results were showed as numbers of F4/80^+^ cells in per mm^2^ mucosa **(C)**. Each bar represents the mean ± SEM; *p < 0.05, **p < 0.01, and ***p < 0.005.

There was constitutive level of macrophages (F4/80^+^ cells) in the lamina propria from GHSR^−/−^ and WT mice without significant difference (Figure 5A and B). Infiltrating macrophages were recruited substantially in the inflamed colon following 7 days DSS challenge from both GHSR^−/−^ and WT mice. Compared to unchallenged, there was a ~5 or ~9 fold increase of colonic F4/80^+^ cell infiltration in GHSR^−/−^ (*p* < 0.01), or WT (*p* < 0.005), respectively. The level of infiltrating colonic macrophages was ~2 fold higher in DSS-treated WT mice compared to GHSR^−/−^ mice at 7 days treatment.

### Lack of GHSR suppresses macrophage cytokine production

**Figure 6:**
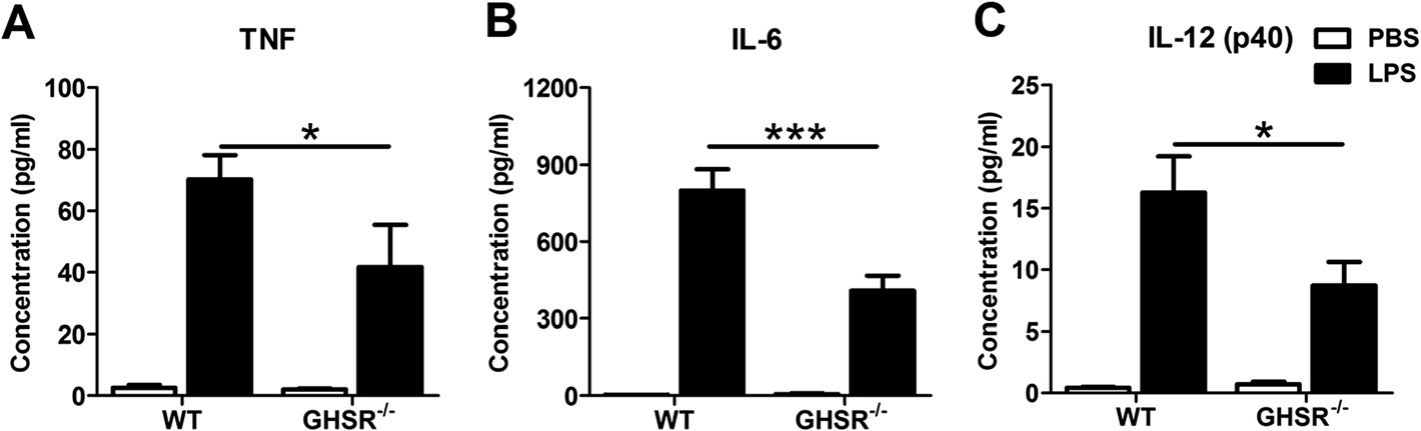
**LPS-induced pro-inflammatory cytokine productions in macrophages from WT and GHSR** ^**−/−**^ **mice.** The primary peritoneal macrophages from WT and GHSR^−/−^ mice were isolated and stimulated with LPS for 2 hours. The productions of pro-inflammatory cytokines, TNF **(A)**, IL-6 **(B)** and IL-12 (p40) **(C)**, were detected in the supernatants by ELISA. Each bar represents the mean ± SEM; *p < 0.05 and ***p < 0.005.

There was a constitutive level of TNF in the supernatant of cultured primary peritoneal macrophages from both GHSR^−/−^ and WT mice. TNF was induced significantly in LPS-stimulated macrophages from GHSR^−/−^ and WT mice (*p* < 0.005). However, the level of TNF from LPS-stimulated GHSR^−/−^ macrophages was significantly lower than that of WT (*p* < 0.05) (Figure 6A). Additionally, similar patterns were observed in IL-6 and IL-12 (p40) productions. The levels of LPS-induced IL-6 or IL-12 (p40) in the supernatant of macrophages from GHSR^−/−^ mice was reduced by ~50% (p < 0.005) and ~35% (p < 0.05) respectively, compared to that of WT mice (Figure 6B, C).

### GHSR antagonist alleviates the cytokines released by macrophages

**Figure 7:**
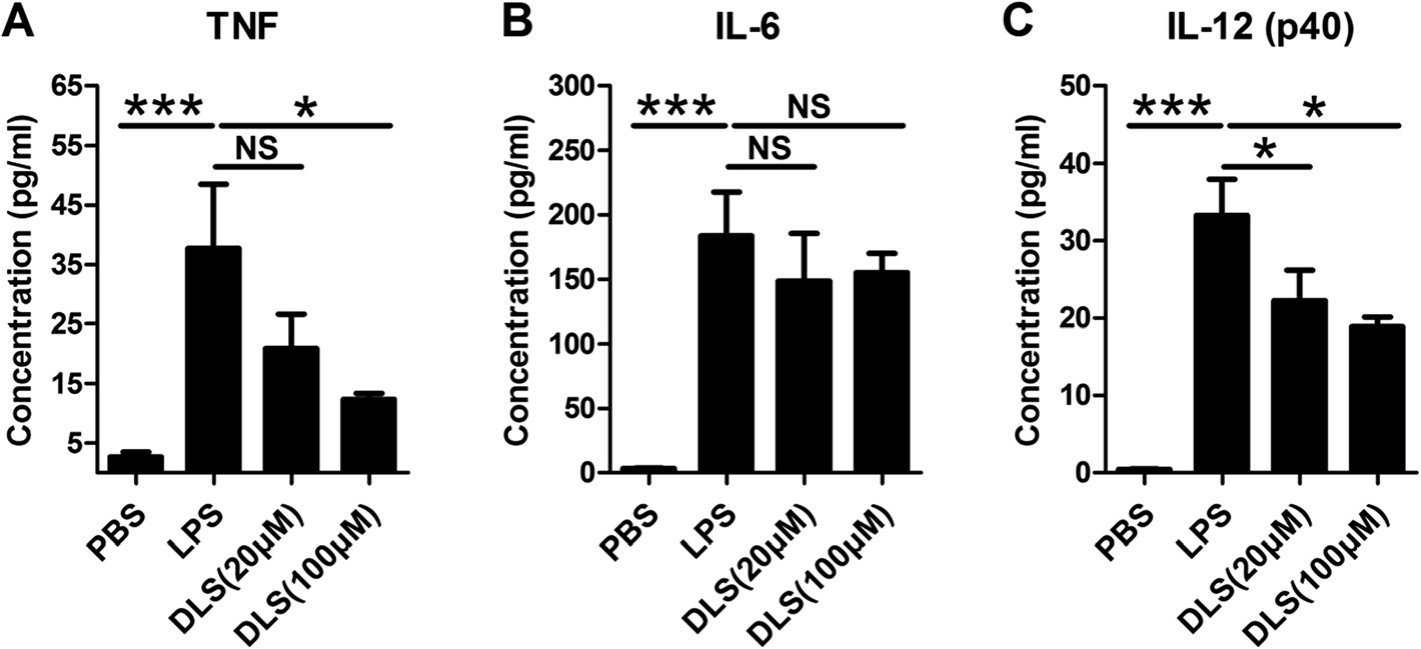
**Effect of GHSR antagonist on LPS-induced pro-inflammatory cytokine productions in macrophages from C57/BL6 mice.** C57/BL6 primary peritoneal macrophages were isolated and stimulated with LPS for 2 hours. Two doses of DLS (20 and 100 μM) were administrated to cells 24 hours before LPS stimulation. The productions of TNF **(A)**, IL-6 **(B)** and IL-12 (p40) **(C)**, were detected in the supernatants by ELISA. Each bar represents the mean ± SEM; NS: no significance, *p < 0.05 and ***p < 0.005.

DLS, an antagonist of GHSR, suppressed the LPS-stimulated WT macrophage TNF production by ~70% (*p* < 0.05) at 100 μM compared to mock treated cells (Figure 7A). Similarly, it was observed that DLS at 20 or 100 μM reduced IL-12 (p40) by >30% or >40%, respectively (*p* < 0.05) (Figure 7C). However, no inhibitory function was observed for IL-6 (Figure 7B).

## Discussions

In the current work, we demonstrated that GHSR regulated the development of acute DSS-induced colitis *in vivo* and participated in macrophages activation *in vitro*. These findings suggest GHSR involvement in the pathogenesis of IBD.

GHSR exhibits a broad tissue distribution in mammals, including various immune organs and cells [3]. In the current study, the expression of GHSR in colon and MLN was upregulated following the progression of DSS-induced colitis, suggesting GHSR contributes to inflammatory response in colon. Additionally, ghrelin expression was also upregulated in MLN, suggesting that GHSR/ghrelin signaling pathway is important in gastrointestinal mucosal inflammation. These findings are supported by clinical observations, showing that the circulating/mucosal ghrelin in IBD patients is significantly higher compared to remission and healthy controls [15,16]. This is also consistent with elevated colonic GHSR expression and the infiltrated numbers of GHSR^+^ T cells from active IBD patients [15].

The role of GHSR was further determined in DSS-induced colitis from GHSR^−/−^ mice and their WT littermates, showing that lack of GHSR significantly ameliorated the severity of acute DSS-induced colitis at clinical, macroscopic and microscopic levels, compared to WT mice. In addition, these clinical data are also in line with a lower spleen weight/size increasing in DSS-treated GHSR^−/−^ mice, which might be due to lower systemic inflammation [13,14]. Interestingly, there was no significant difference in body weight loss between both genotypes, which might be due to the acuteness of DSS-induced colitis. Our results demonstrated amelioration of colon shortening in GHSR^−/−^ mice compared to WT mice following 7 days of DSS challenge, suggesting GHSR plays a key role in the development of acute colitis. Moreover, these data are also in line with evidence from histopathology. Our current finding is supported by others, showing that absence of ghrelin attenuates DSS-induced colitis, and exogenous treatment with ghrelin enhances the manifestations of disease [10].

IBD is one of the fields where the immune regulatory effect of ghrelin/GHSR is of considerable relevance. DSS-induced acute colitis mainly relates to innate immunity [9,11], and TLRs play an important role in innate immunity. Exaggerated TLR signaling might contribute to pathogenesis of IBD [17]. The present study demonstrated that colonic and MLN expression of TLR-2 or TLR-4 in DSS treated GHSR^−/−^ mice was significantly lower than that of WT. This observation suggests that GHSR contributes to colonic inflammation *via* regulating TLRs mediated innate immunity.

The profile of pro-inflammatory cytokines is a general standard that reflects colonic inflammatory status of IBD patients and animal colitis models. In the present study, we observed reduced colonic pro-inflammatory cytokines both at protein and RNA levels from DSS-challenged GHSR^−/−^ mice compared to that of WT. This observation further supports the important role of GHSR in colonic inflammation.

Furthermore, M-CSF/GM-CSF are critical modulators in regulating myeloid cells proliferation, maturation and recruitment, which might implicate them in the pathogenesis of IBD [18]. M-CSF gene deficiency or blockage attenuates DSS-induced colitis, accompanied with less infiltration of immune cells [19,20]. Our data showed that M-CSF and GM-CSF were also lower expressed in DSS-treated GHSR^−/−^ mice, compared to that of WT. This is also consistent with reduced colonic macrophage infiltrations in DSS-challenged GHSR^−/−^ than WT. GM-CSF contributes to neutrophil accumulation and priming in IBD [21], with elevated GM-CSF in mucosal lesions from IBD patients and mice colitis models [13,21]. We have demonstrated that colonic GM-CSF expression was significantly induced in DSS-treated WT mice, but not GHSR^−/−^, compared to that of mock challenge, suggesting that GHSR mediated colonic inflammation might be *via* M-CSF and GM-CSF.

Additionally, significantly reduced pro-inflammatory cytokines [TNF, IL-6 and IL-12 (p40)] were observed in LPS-stimulated macrophages from GHSR^−/−^ mice compared to WT *in vitro*, suggesting GHSR plays a key role in macrophage activation. This is also confirmed by the finding that DLS, a GHSR antagonist, suppressed LPS-induced pro-inflammatory cytokine releases in macrophages from WT mice.

In the current work, we found that GHSR regulates immune reaction. However, exogenous ghrelin is considered as an anti-inflammatory modulator *in vitro* and *in vivo* [5,12]. Such discrepancy might be due to endogenous and exogenous conditions and/or different animal models. Moreover, ghrelin is an important hormone with various functions on other systems, e.g. stimulating GH/IGF-1 axis and gut motility [22,23], which might also contribute to the pathogenesis of IBD [22,24]. In addition, the role of ghrelin/GHSR in intestinal epithelium might be pro-inflammatory, as IL-8 is up-regulated in GHSR over-expressing colonic epithelial cells with ghrelin stimulation [7].

## Conclusions

In summary, gastrointestinal GHSR and ghrelin were induced in response to DSS stimulation. DSS-induced colitis was ameliorated in GHSR^−/−^ mice compared to WT. The possible mechanism may be mediated by inhibiting innate immunity, leading to a reduction of pro-inflammatory cytokine productions and a decrease of local macrophage infiltration *in vivo* and *in vitro*. Our data demonstrated that GHSR contributes to the development of DSS-induced colitis, and suggest GHSR as a potential therapeutic target for IBD.

## Materials and methods

### Animals

In the experiment of temporal expression patterns, 8 week-old male C57/BL6 mice were selected. For DSS-induced colitis, GHSR^−/−^ mice and their WT littermates were generated as described previously [25]. All animals were supplied by *Shanghai Research Centre for Model Organisms* and housed under specific pathogen-free environment with food and water *ad libitum*. The environment was maintained at 22°C with a 12 h light/dark cycle. Animal welfare and experimental procedures were carried out strictly in accordance with the guidance for care and use of laboratory animals (*National Research Council of USA, 1996*) and approved by *Institutional Animal Care and Use Committee of Shanghai Research Centre for Model Organisms*.

### Experimental colitis

Mice were customised environment one week in the animal house prior to DSS challenge. Acute colitis was induced by giving 2% DSS (MW: 36 ~ 50 k; MP Biomedicals, Australia) in normal drinking water for 7 days as described previously [13]. Mice were allowed free access to water and food during experiments.

For temporal expression pattern analysis, C57/BL6 mice were sacrificed at 0, 4 and 7 days post DSS challenge (n = 3 for each time point). The colon, mesenteric lymph node (MLN) and spleen were collected and snap frozen in liquid nitrogen.

For determining the role of GHSR in the development of colitis, body weight and fecal score (consistency and stool blood) were determined daily post DSS challenge in both GHSR^−/−^ and WT mice (control: n = 8, DSS: n = 11). Body weight change of animals from different genotypes/treatments was expressed as percent body weight gain/loss, in comparison with day 0. Disease activity index (DAI) [14] consists of the following parameters: body weight loss (0 ~ 4), stool consistency (0 ~ 4) and fecal blood (0 ~ 4) were monitored. Colons and MLN were collected at day 7 post DSS challenge, and the lengths of colon and weights of spleen were measured.

For understanding the experimental procedures, a flow diagram was provided in the Additional file 1: Figure S1.

### Histological assessment

Colons were harvested from GHSR^−/−^ mice and their WT littermates, as “Swiss roll” [26]. The rolls were fixed in 4% paraformaldehyde over night for tissue processing and wax embedding. The colon was sectioned (5 μm) for haematoxylin and eosin (H&E) staining and immunohistochemistry.

Histopathology was scored by an independent pathologist, as described previously [13]. Two independent parameters were measured: the extent of inflammation (0, none; 1, slight; 2, moderate; 3, severe; 4, massive) and the extent of crypt damage (0, none; 1, the basal one-third portion damaged; 2, the basal two-thirds portion damaged; 3, the entire crypt damaged but the surface epithelium intact; 4, the entire crypt and epithelium lost). The three portions of colon (ascending, transverse and descending colon) were scored respectively.

### Immunohistochemistry for macrophages

For macrophages identification, immunohistochemical staining of F4/80 (1:400, Abcam, USA) was processed as described previously [13], which is a broadly used cell marker for macrophages. The binding of the primary antibody was detected with a biotin-conjugated secondary antibody (1:200, Biolegend, USA) followed by a streptavidin-HRP reaction. Diaminobenzidine (DAB) was used as the immunodetection substrate.

### Colonic cytokines determination

Colon samples were homogenised in RIPA lysate (Thermo Fisher Scientific, USA), and the supernatant was collected for assessments of pro-inflammatory cytokines (n = 4 for each group). TNF and IL-6 were detected using the commercial ELISA kits (Biolegend, USA), according to the instruction from the manufacturer. The final results were represented as the ratio of cytokine concentration to total protein content, which was determined using a BCA reagent kit (Beyotime Institute of Biotechnology, China).

### Gene expression analysis

The temporal expressions of GHSR/ghrelin responding to DSS challenge were detected in colon, MLN and spleens, respectively. Moreover, colon and MLN from mock or DSS-challenged GHSR^−/−^ and WT mice, were harvested for detecting the RNA levels of TNF, IL-1β, IL-6, IFN-γ, M-CSF, GM-CSF, TLR-2 and TLR-4. Total RNA in tissue samples was isolated using Trizol (Roche Diagnostics, Germany) and reverse transcribed by an iScript cDNA Synthesis Kit (Bio-Rad, USA). The gene expressions were quantified by rRT-PCR, using an iQ SYBR Green Supermix (Bio-Rad, USA) and a gradient cycler machine (Eppendorf, Hamburg, Germany). β-actin was chosen as the reference gene. Data was represented as 2^–ΔΔCT^. All primer sequences were showed as follow:

- Ghrelin: 5′-CCATCTGCAGTTTGCTGCTA-3′ (forward), 5′-TGACAGCTTGATGCCAACAT-3′ (reverse);
- GHSR: 5′-AGATCGCGCAGATCAGTCAG-3′ (forward), 5′-GTATTGATGCTCGACTTTGTCCA-3′ (reverse);
- TNF: 5′-CCTGTAGCCCACGTCGTAG-3′ (forward), 5′-GGGAGTAGACAAGGTACAACCC-3′ (reverse);
- IFN-γ: 5′-ATCTGGAGGAACTGGCAAAA-3′ (forward), 5′-TGAGCTCATTGAATGCTTGG-3′ (reverse);
- IL-1β: 5′-5′-GAAATGCCACCTTTTGACAGTG-3′ (forward), 5′-TGGATGCTCTCATCAGGACAG-3′ (reverse);
- IL-6: 5′-ACAACCACGGCCTTCCCTACT -3′ (forward), 5′-GCCATTGCACAACTCTTTTCTCAT-3′ (reverse);
- M-CSF: 5′-GTGTCAGAACACTGTAGCCAC-3′ (forward), 5′-TCAAAGGCAATCTGGCATGAAG-3′ (reverse);
- GM-CSF: 5′-GGCCTTGGAAGCATGTAGAG-3′ (forward), 5′-CCGTAGACCCTGCTCGAATA-3′ (reverse);
- TLR-2: 5′-GCAAACGCTGTTCTGCTCAG-3′ (forward), 5′-AGGCGTCTCCCTCTATTGTATT-3′ (reverse);
- TLR-4: 5′-ATGGCATGGCTTACACCACC-3′ (forward), 5′-GAGGCCAATTTTGTCTCCACA-3′ (reverse);
- β-actin: 5′-ATTGCTGACAGGATGCAGAA-3′(forward), 5′-GCTGATCCACATCTGCTGGAA-3′ (reverse).

### Isolation of peritoneal macrophages and stimulation with LPS

Peritoneal macrophages from GHSR^−/−^ and WT mice were collected as described before [27]. In brief, cells were harvested *via* peritoneal lavage with 5 ml RPMI 1640 medium (Invitrogen, USA). Red blood cells were removed by red blood lysis buffer (Beyotime Institute of Biotechnology, China), and the peritoneal cells were seeded into a 12-well plate at 2 × 10^6^ cells/well and incubated at 37°C with 5% CO_2_ for 2 hours. After removing non-adherent cells by washing twice with PBS, the adherent macrophages were supplemented with fresh RPMI 1640 medium containing 10% fetal calf serum (FCS).

Activation of macrophage was induced by 100 ng/ml LPS (Sigma-Aldrich, USA) for 2 hours incubation. Furthermore, to confirm whether GHSR is involved in the function of macrophage, peritoneal macrophages were collected from C57/BL6 mice, and given DLS (Bachem, UK) at 20 or 100 μM after LPS 24 hour stimulation. The levels of TNF, IL-6 and IL-12 (p40) in culture supernatants were determined by ELISA kits (Biolegend, USA).

### Statistical analyses

All data are shown as mean ± SEM. Differences between groups were estimated using one-way or two-ways analysis of variance (ANOVA), followed by the Bonferroni posttest analysis. Significant differences were accepted when *p* values <0.05. The statistic analysis was calculated and plotted using GraphPad Prism version 4.0 (GraphPad Software, Canada).

## Additional file

Additional file 1: Figure S1. The diagram demonstrated the experimental procedure.

## Abbreviations

GHSR: Growth hormone secretagogue receptor
GH: Growth hormone
DLS: D-lys3-GHRP6
IBD: Inflammatory bowel disease
CD: Crohn’s disease
UC: Ulcerative colitis
MLN: Mesenteric lymph node
DAI: Disease activity index
H&E: Haematoxylin and eosin
DAB: Diaminobenzidine
TNF: Tumor necrosis factor
IFN-γ: Interferon gamma
IL: Interleukin
M-CSF: Macrophage colony stimulating factor
GM-CSF: Granulocyte-macrophage colony stimulating factor
TLR: Toll-like receptor
FCS: Foetal calf serum
LPS: Lipopolysaccharide
PBS: Phosphate buffered saline
ANOVA: Analysis of variance
