# Supplementary figures and images for: Growth hormone secretagogue receptor is important in the development of experimental colitis
Source: Cell Biosci. 2015 Mar 21;5:12. doi: 10.1186/s13578-015-0002-5 (PMC4377856; doi:10.1186/s13578-015-0002-5)

## Slide 1
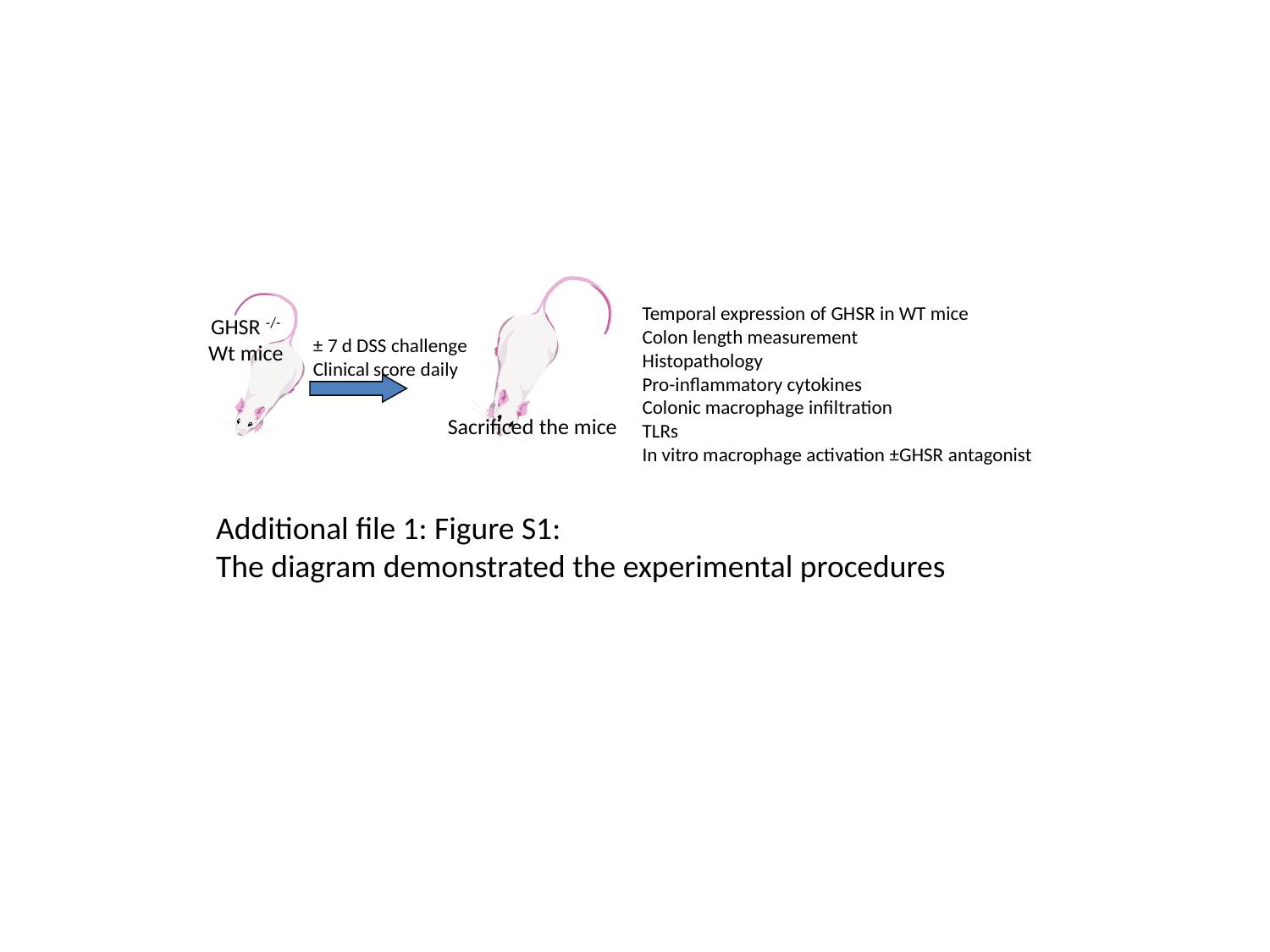

Additional file 1: Figure S1:
The diagram demonstrated the experimental procedures

Supplement: Additional file 1: Figure S1. — The diagram demonstrated the experimental procedure. [file 13578_2015_2_MOESM1_ESM.ppt]
